# Skin conductance variability in ICU patients: an observational study of the relation to pain and Motor Activity Assessment Scale level

**DOI:** 10.1186/cc9776

**Published:** 2011-03-11

**Authors:** AC Günther, AR Schandl, H Storm, P Rossi, PV Sackey

**Affiliations:** 1 Karolinska Institutet, Stockholm, Sweden; 2 Rikshospitalet University Hospital, Oslo, Norway

## Introduction

Many patients describe pain and other adverse feeling from their ICU stay and the impact of such feelings impacts long-term psychological morbidity. Presently no objective method for detecting pain or distress is available. Skin conductance variability has been investigated as a monitor of perioperative pain. The method has not been studied in adult ICU patients.

## Methods

Twenty-five (13 intubated and 12 non-intubated) patients were included in this observational study. Patients were monitored with the MED-STORM Stress Detector for 1 hour of intensive care treatment and care. Skin conductance variability (number of skin conductance fluctuations per second (NSCF)) was measured and patients were observed in parallel during rest and during procedures and staff-patient interactions. The sedation-agitation level was monitored with the Motor Activity Assessment Scale. Pain was monitored with the Numeric Rating Scale (0 to 10) in communicating patients and by observation of expressions of pain in patients unable to communicate verbally.

## Results

In non-intubated patients, NSCF values were low when patients were unstimulated and comfortable and increased with increasing stimulation but also with increasing agitation without any apparent pain. The highest NSCF values were noted during combined pain and agitation. In intubated patients, a similar pattern was observed but with generally lower values, most likely due to sedation. Sensitivity and specificity of NSCF at a cut-off value > 0.13 for detecting expressed pain/discomfort were 74% and 55% for non-intubated patients and 61.5% and 68% for intubated patients.

## Conclusions

Skin conductance variability increases in critically ill patients with increasing stimulation but is also affected by the level of sedation/agitation, making the method unsuitable for detecting pain alone in critically ill patients, but possibly of value to more generally monitor emotional stress with different etiology. Further studies of the method in critically ill patients, over longer time and with validated pain instruments are warranted.

## Conflicts of interest

HS is a co-owner of Med-Storm AS, the company responsible for the production and distribution of the Med-Storm Stress Detector. The other authors declare that they have no conflicts of interest.

